# Clinical Efficacy of Intravenous Immunoglobulins in Management of Toxic Shock Syndrome: An Updated Literature Review

**DOI:** 10.7759/cureus.12836

**Published:** 2021-01-21

**Authors:** Sana Amreen, Simrandeep K Brar, Sumera Perveen, Muhammad Reza Chaudhry, Sarah AlBabtain, Safeera Khan

**Affiliations:** 1 Internal Medicine, California Institute of Behavioral Neurosciences & Psychology, Fairfield, USA; 2 Internal Medicine/Family Medicine, California Institute of Behavioral Neurosciences & Psychology, Fairfield, USA; 3 Family Medicine, Ibne Sina Hospital Parco MCR, Multan, PAK; 4 Psychiatry, California Institute of Behavioral Neurosciences & Psychology, Fairfield, USA; 5 Public Health and Preventive Medicine, St. George's University School of Medicine, St. George's, GRD; 6 Psychiatry and Behavioral Sciences, California Institute of Behavioral Neurosciences & Psychology, Fairfield, USA

**Keywords:** toxic shock syndrome (tss), intravenous immunoglobulins (ivig), streptococcal tss, staphylococcal tss, and treatment of tss, management of toxic shock syndrome

## Abstract

Toxic shock syndrome (TSS) is an uncommon complication of infection caused by streptococci and staphylococci. It is associated with a high mortality rate. When evaluating patients with shock symptoms from skin or soft tissue sources, a high index of suspicion for TSS must be maintained. Prompt diagnosis and integrative management with surgical intervention, antibiotics, hemodynamic stabilization, and adjuvants like intravenous immunoglobulins improve survival.

## Introduction and background

Toxic shock syndrome (TSS) is a toxin-mediated disease, most commonly caused by invasive Group A streptococcal (GAS) and staphylococcal infections leading to immune activation and massive cytokine release. While streptococci are well known to cause a range of infections from benign pharyngitis to more serious conditions like endocarditis, scarlet fever, pneumonia, meningitis, osteomyelitis, rheumatic fever; skin infections like cellulitis, necrotizing fasciitis, myositis, and bacteremia; and septic shock [1], Streptococcal TSS may result from any condition caused by the streptococci. Staphylococci are the normal commensals in skin and nares and are commonly known to cause skin and soft tissue infections in patients with colonization [2]. Bloodstream infection, pneumonia, and Methicillin-resistant *Staphylococcus aureus *(MRSA) infections are more commonly seen in hospitalized patients. The use of tampons, menstrual cups, and nasal packing are considered risk factors for staphylococcal TSS [3]. 

Toxic shock syndrome carries a very high mortality rate and is thought to have three phases in its pathogenesis [1]. It is associated with rapid onset of action with symptoms like high fever, hypotension, multi-organ failure, and erythematous rash. TSS's exact pathogenesis is unclear, but studies have shown a complex interplay between bacterial toxins and the body's response to the infection as the cause of the severity of the clinical manifestations seen [1]. The toxins that cause TSS are referred to as superantigen and include staphylococcal enterotoxins, toxic shock syndrome toxin-1 (TSST-1), and streptococcal pyrogenic exotoxins [4]. They are generated by toxigenic strains of *Streptococcus pyogenes *and *Staphylococcus aureus *that have acquired an underlying genetic material needed to transcribe the toxins from a plasmid or a bacteriophage. The superantigen binds and forms the superantigen-major histocompatibility (MHC) class-II complex with the MHC class-II, which then binds to T cell receptors, leading to non-specific activation of T cells, leading to a massive release of pro-inflammatory cytokines, which are responsible for the systemic toxicity. Prompt diagnosis and management of TSS is the key component in patient survival. Treatment consists of supportive care and antibiotics like clindamycin, which is mostly bacteriostatic through bacterial 50S ribosomal subunit binding. The use of intravenous immunoglobulins (IVIG) plays a vital role in neutralizing cytokines. One study has shown some difference in the disease severity and the efficacy of IVIG in the treatment of TSS in children compared to adults [5]. There is a paucity of clinical trial data regarding the role of IVIG in TSS treatment in children and adults. Our review aims to consolidate the existing knowledge on the pathogenesis, clinical features, and TSS management. We aim to shed light on IVIG's use in its treatment, as it remains a serious and life-threatening condition. 

## Review

Method

We performed a comprehensive data search using online databases like PubMed and Google scholar. The keywords used for our search included Toxic Shock Syndrome (TSS), IVIG, streptococcal TSS, staphylococcal TSS, and treatment of TSS. They were used alone and in combination. The initial search resulted in 176 studies; after adding filters, we narrowed the results down to 62 studies. After our final scan, we included 31 studies as a part of our review. We did not perform a quality assessment. 

*Inclusion Criteria: *Based on their title and abstract content, we include the studies relevant to our research question for our final review. The studies included are clinical trials, case studies, case reports, meta-analysis, randomized controlled trials, reviews, and systematic reviews from geographical locations worldwide. Our studies included articles in the English language and *in vitro *and *in vivo *studies performed on humans. The time frame for our studies included articles from the time of inception till 2020. While some of them included full text, the others included abstracts. 

*Exclusion Criteria: *We did not include published studies in languages other than English. We did not have editorial letters. Studies that included animal research were not a part of our review.

**Figure 1:**
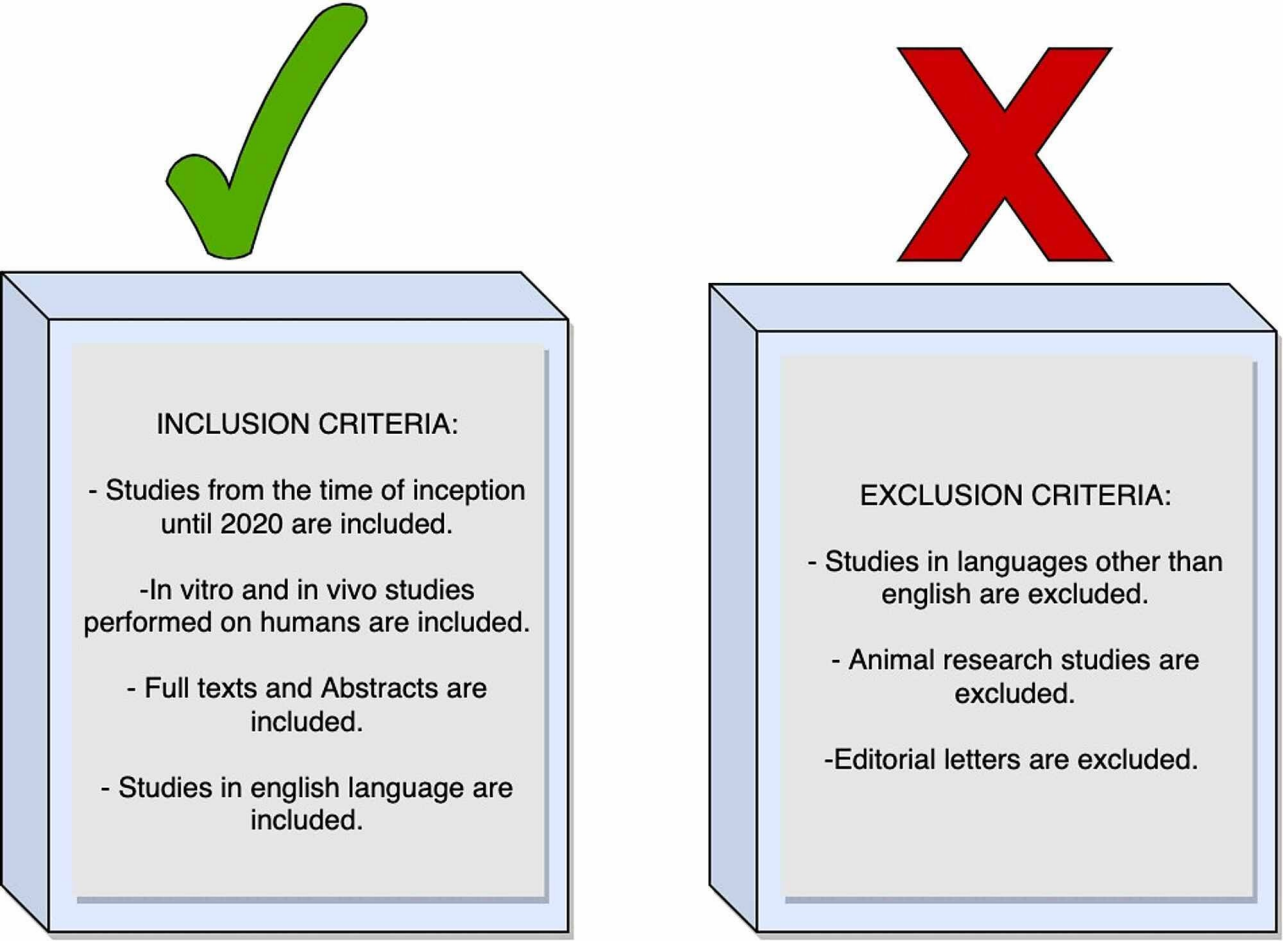
Displaying the inclusion and exclusion criteria

**Figure 2:**
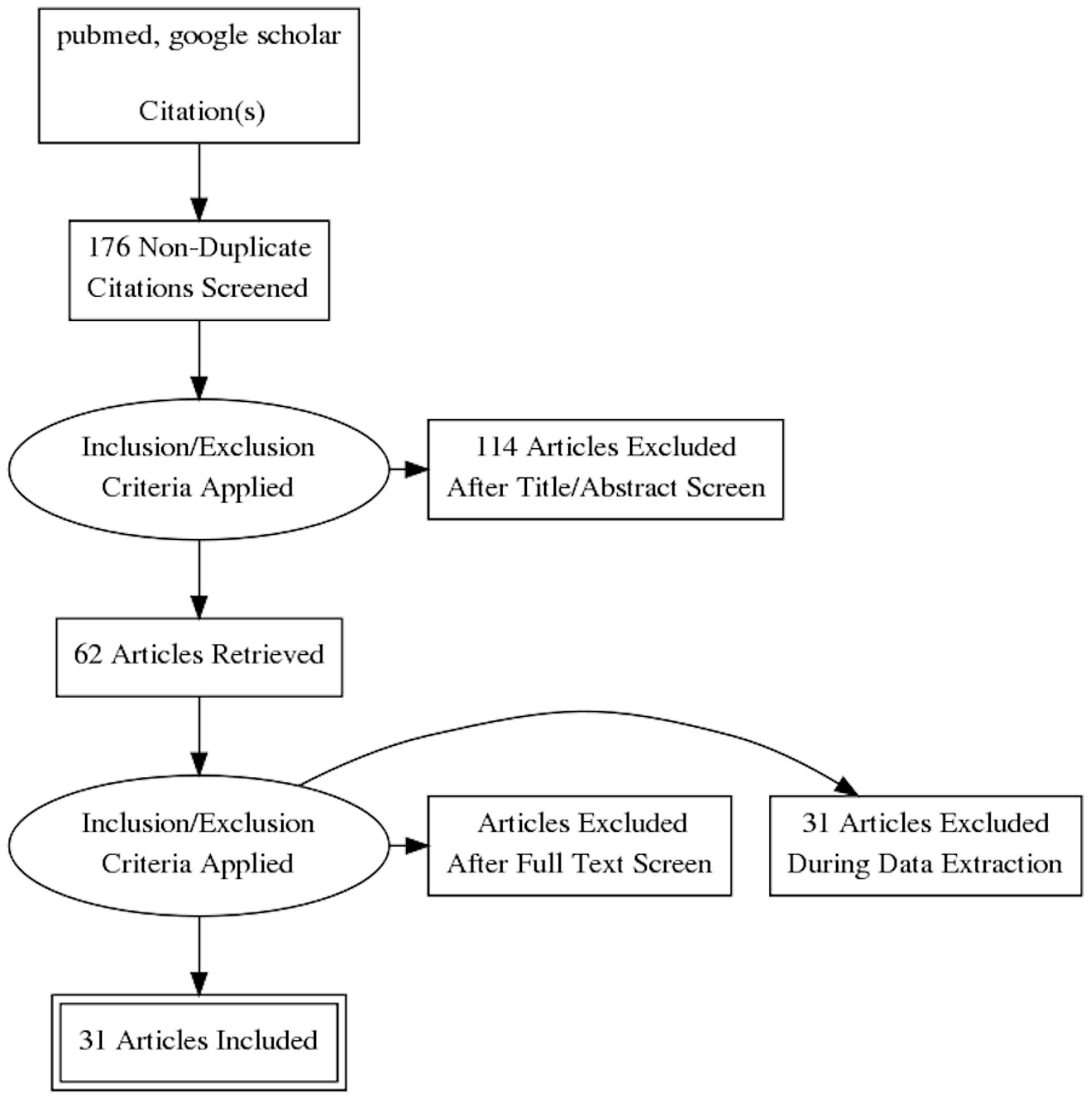
Prisma diagram for the process of collection of studies

Figure 1 shows the inclusion and exclusion criteria. Figure 2 shows the process of collection of studies in a PRISMA diagram. 

Discussion

Toxic shock syndrome is a rare complication of bacterial infection most commonly associated with Group A streptococcus and staphylococcus and is notoriously known for its high mortality rate. A study performed in the United Kingdom discussed unusual Group D streptococci as TSS's cause in three patients [6]. Data collected from the Centers for Disease Control and Prevention (CDC) showed an annual incidence of 0.2 per million inhabitants with a case fatality rate as high as 36% [7]. While some studies suggest an equal incidence in all age groups, another study states a lower incidence in children than adults [8]. Data collected from our studies have shown its association in patients with comorbid conditions and worse prognosis seen in individuals of extreme ages, chronic conditions like diabetes, alcohol abuse, weakened immune system, and those with delayed diagnosis [7,9,10].

*Pathophysiology: *The term 'Superantigen' means an antigen that can overstimulate the immune system. The toxic shock syndrome's primary pathophysiology is based on toxin’s expression by the invasive bacterial strains, which act as a superantigen and cause non-specific activation of T cells. This activation then leads to a massive release of inflammatory cytokines and their detrimental sequelae, including circulatory failure.

Typically, the antigen-presenting cells (APCs) engulf the foreign particles like bacteria and degrade them in the phagolysosome and load their partially degraded peptides on the major histocompatablity complex (MHC) class-II, which is expressed on the surface of the APC. The loaded MHC class-II binds at the antigen grove present on the surface T-cell receptor (TCR), causing activation of the T cells, which generates a monoclonal T-cell-mediated response that is specific against the antigen which has been presented. 

**Figure 3:**
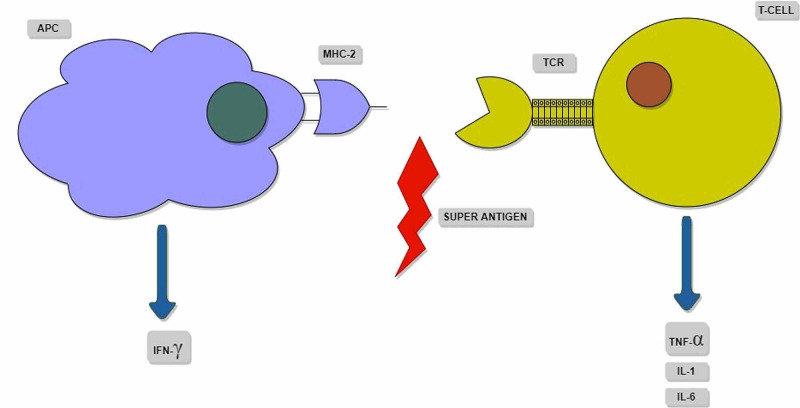
Schematic representation of the superantigen binding outside the peptide grove, causing non-specific T- cell activation. APC: antigen-presenting cell; MHC-II: major histocompatibility complex-II; TCR: T-cell receptor; IFN: interferon; TNF: tumor necrosis factor; IL: interleukins

In toxic shock syndrome, the superantigen cross-links the TCR and the MHC class-II outside the standard peptide-binding groove with high affinity. This cross-linkage acts like an activating signal for the T cells, which activates almost 40% of the naïve T cells [11]. This causes a non-specific, polyclonal response, resulting in a massive release of interferon-gamma, which in turn activates the macrophages, which results in overexpression of pro-inflammatory cytokines like interleukin (IL)-1, IL-6, and tumor necrosis factor-alpha. This rapid surge of cytokines causes a capillary leak, hypotension, and circulatory failure. The superantigen-activated T cells activate the inflammatory and the coagulation pathway leading to a rapid clinical deterioration [11]. The pathophysiology of non-specific T-cell activation by superantigens binding outside the peptide groove is shown in Figure 3.

*Clinical Features: *A study performed in Yorkshire described the clinical features of toxic shock syndrome in three stages [12]. In the first stage, the symptoms are vague and non-specific, making the diagnosis difficult; having a defined portal of entry for infection like a cut or wound aids in the diagnosis. The only prominent symptom at this stage is pain out of proportion to the examination. Treatment of TSS during this stage with antibiotics is easier as complex sequelae are typically absent. The second stage is marked by prominent symptoms and signs like fever, body aches, nausea, vomiting, diarrhea, cardiovascular instability characterized by fluctuating hypertension, and hypotension. At this stage, the toxin-induced cytokine storm is established [13]. Although the clinical picture indicates TSS's possibility at this stage, having visible signs of infection or portal of entry aids in definitive diagnosis. Supporting lab evidence that suggests TSS as the most probable diagnosis at this stage are elevated creatinine phosphokinase, elevated creatinine, reduced serum albumin, and slightly reduced serum bicarbonate [12]. Treatment at this stage requires empiric broad-spectrum antibiotics, but cytokine neutralization is essential in halting the clinical deterioration and rapid system failure. intravenous immunoglobulins (IVIG) are used to neutralize the cytokines.

The third stage is associated with a worse prognosis and the highest mortality rate. It is characterized by widespread bacteremia, sepsis, systemic shock, and multi-organ failure. Desquamation, visible ecchymosis, bullae, and edema are also notable findings. CT scan and MRI can help identify any deep infection source when the visible portal entry is not apparent [1]. Treatment at this stage comprises supportive care for the failing organs. Dialysis, mechanical ventilatory support, and aggressive surgical debridement are necessary, along with the existing treatment of broad-spectrum antibiotics and IVIG. A study discusses the use of clindamycin as a preferred antibiotic because of its ability to suppress bacterial toxin production [12].

**Figure 4:**
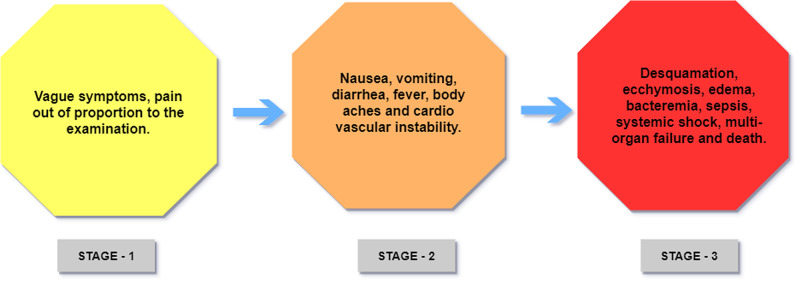
Different stages in the clinical features of Toxic Shock Syndrome

A few studies have shown an association between non-steroidal anti-inflammatory drugs (NSAIDs) use before developing TSS [8,12]. The researchers also report invasive Group A streptococcal (GAS) infection in children following varicella infection, which can cause TSS. A study performed in the United States in children displayed a worse prognosis with streptococcal TSS when compared to staphylococcal TSS [14]. Figure 4 shows various stages in the clinical manifestation of toxic shock syndrome.

*Treatment: *The mainstay of treatment depends on the time of diagnosis. Intensive care unit admission is required in most cases. Antibiotics are the first line of treatment for TSS in its early stages as it kills the bacterial colony responsible for the toxin production. Later stages are difficult to treat. We discuss IVIGs in our studies to neutralize the toxins. They require aggressive fluid resuscitation and supportive care for the failing organs.

Antibiotics*: *Penicillin containing beta-lactam antibiotics are effective against streptococcus and staphylococcus. Clindamycin works primarily by binding to the 50s ribosomal subunit of bacteria and it is not only is bacteriostatic but also inhibits toxin production. Therefore, it is used as the drug of choice in TSS and has been discussed in a few studies as a preferred antibiotic of choice [12,15-17]. 

IVIG: Various studies have discussed using intravenous immunoglobulins to treat various critical conditions with multi-system involvement like septic shock, immunodeficiency disorders, disseminated infections like toxic shock syndrome, and complicated *Clostridium difficile *infections [18,19], which does not respond to conventional treatment. Immunoglobulins can be monoclonal or polyclonal [20]. A systematic review performed on the use of IVIG explained the superior efficacy of polyclonal IVIG in the treatment of TSS [21-23]. Moreover, human-derived poly immunoglobulins demonstrated superior neutralizing power compared to synthetic and animal-derived immunoglobulins. Mechanistically, immunoglobulins work by neutralizing the superantigen, which is the source of toxin production [24,16]. IVIG has shown similar efficacy in the treatment of both staphylococcal and streptococcal TSS [25]. Some studies have also discussed close contact prophylaxis with antibiotics as a part of TSS management [26].

*Side Effects*: Immunoglobulins are relatively safe to use in most patients, but they do carry a risk of unwanted side effects. A study discussed these common side effects and may range from benign transfusion reactions to severe conditions like kidney failure, aseptic meningitis, and thromboembolic events [27]. 

*Adjuvants*: One study reviewed by us also mentioned hyperbaric oxygen therapy as an adjuvant along with IVIG in the treatment of severe diseases, including TSS [28]. In a study performed in Australian children, fresh frozen plasma was used as a part of treatment, but its exact therapeutic benefit is unclear. Patients with severe TSS and organ damage like renal failure and lung injury require dialysis and mechanical ventilation.

Table 1 summarizes the studies that advocated IVIG's efficacy in treating streptococcal and staphylococcal TSS in children and adults.

**Table 1:** Summary of the studies advocating the efficacy of IVIG in treating staphylococcal and streptococcal TSS in children and adults TSS: toxic shock syndrome; IVIG: intravenous immunoglobulins; GAS: Group A streptococci; ICU: intensive care unit

Author Name	Year of Publication	Type of Study	Purpose of the Study	Results/Conclusions
Schmitz M, et al. [1]	2018	Review Article	To study pathophysiology, clinical features, and management of TSS	The study concluded that the use of IVIG is still not well established, and the decision is made on a case by case basis.
O'Loughlin RE et al. [7]	2007	Review	To determine the cause of TSS and its prevention strategies.	The study determined GAS as the most common cause of TSS, and they discussed introducing a vaccine against it.
Stevens DL [12]	2002	Editorial Review	It was performed to review the cases of TSS in Yorkshire	The study emphasized the development of new diagnostic techniques and management of TSS
Nonfoux L, et al. [3]	2018	In Vitro Study	This study was performed to determine the exact role of tampons and menstrual cups in causing TSS.	They concluded that TSS's risk using tampons and menstrual cups is increased in women with staphylococcal colonization.
Chuang YY, et al. [8]	2012	Review Article	To determine the incidence and risk factors of TSS in the pediatric population	They determined that the overall incidence of both streptococcal and staphylococcal TSS is lower in children. Cases of streptococcal TSS has been reported following varicella infection
Kaul R, et al. [29]	1999	Observational Study	To determine the effectiveness of IVIG in the management of TSS	They concluded that IVIG has proven toxin neutralizing activity and can be used as an adjuvant in the treatment of TSS
Cone LA, et al. [20]	2009	Case Report	To discuss the management of TSS	The study discussed the use of three-drug therapy in the management of TSS with an antibiotic (ceftriaxone), IVIG, and Activated Protein C
Shah PJ, et al. [18]	2015	Review	To determine the need for IVIG in the management of TSS	The study concluded that the IVIG could be used as an adjuvant to manage severe TSS cases, but few clinical trials were performed to back up the claim.
Gaensbauer JT, et al. [14]	2018	Review Article	To determine the contribution of TSS in the pediatric population with septic shock.	The study emphasized having TSS as a possible cause of septic shock in the pediatric population.
Burnham JP, et al. [10]	2018	Review	To discuss the complications and management of severe skin and soft tissue infections.	The study concluded that surgical intervention is required to manage severe skin and soft tissue infections and the use of IVIG as an adjuvant.
Low DE [13]	2013	Review	To discuss the lack of treatment options in the management of TSS	It concluded that the use of IVIG along with extensive surgical intervention reduces morbidity and mortality associated with TSS
Linnér A, et al. [16]	2014	Observational study	To observe the efficacy of IVIG as an adjuvant in the management of TSS	The study concluded that there is improved survival in patients with TSS with the use of IVIG and clindamycin in its management
Cawley MJ, et al. [30]	2012	Case Report and Review	To discuss the management of TSS and explore the use of IVIG in its treatment.	They concluded that IVIG is useful in the treatment of TSS associated with necrotizing fasciitis.
Chen KY, et al. [31]	2016	Retrospective Review Study	To study the pathogenesis, clinical features, and management of TSS in Australian children.	It was determined that early diagnosis and treatment in the ICU with clindamycin and IVIG demonstrated better outcomes.
Wang J, et al. [27]	2015	Literature review	To determine the effectiveness of IVIG as a therapeutic agent.	The study concluded that IVIG could be used in the treatment of TSS along with other infectious and non-infectious conditions
Norrby-Teglund A, et al. [23]	2009	Research article	To determine the cause of TSS and discuss its current management and adjuvant therapies.	The study discussed using IVIG as an adjuvant to treat TSS associated with severe group A streptococcal infections.
Wilkins AL, et al. [26]	2017	Review	To discuss various management options for TSS.	The study discussed the management of TSS and recognized the use of IVIG as an adjuvant.

Limitations

We could not make an exact comparison of IVIG's clinical efficacy in the treatment of TSS in children and adults. Although some studies suggested that streptococcal infection can occur in the community as an outbreak [28], there are insufficient studies to support the claim. There are no studies performed on the global solution to reduce the incidence of streptococcal and staphylococcal infections around the world. We could not conduct a full review of some studies whose full text was unavailable.

## Conclusions

Our review highlighted the clinical efficacy of intravenous immunoglobulins in treating toxic shock syndrome. We discussed pathophysiology, clinical features, and the treatment of toxic shock syndrome, and by doing so, we consolidated the existing knowledge about the topic. We attempted to determine the difference in the epidemiology of TSS in pediatric and adult populations. We could not determine a clear distinction. The risk factor in children includes recent varicella infection. Alcohol abuse, chronic diseases, use of nasal packing and menstrual cups and tampons, and immunocompromised states are the risk factors seen in adults. Others can explore more treatment options in managing toxic shock syndrome, and the existing research done on this topic is insufficient.

We conclude that toxic shock syndrome is a fulminant, toxin-mediated disease, most commonly caused by invasive Group A streptococcal (GAS) and staphylococcal infections leading to immune activation and massive cytokine release, resulting in the rapid clinical deterioration with cardiovascular instability, bacteremia, sepsis, multi-organ failure, and death. When evaluating patients with shock symptoms from skin or soft tissue sources, a high index of suspicion for TSS must be maintained, as timely diagnosis and treatment with antibiotics like clindamycin, IVIGs, aggressive fluid resuscitation, and supportive care to the failing organs can reduce the mortality rate. IVIG neutralizes the superantigen and is effective in both streptococcal and staphylococcal TSS. Table 1 displays 17 studies that advocated the efficacy of IVIG. There are a few studies that could not draw a conclusion regarding the efficacy of IVIG. However, we conclude that IVIG can be accepted as an adjuvant treatment option in the management of TSS as it neutralizes the superantigen and halts the cytokine production responsible for the clinical deterioration seen in TSS.
